# Biodegradable Starch-Based Films Incorporating Banana and Orange Waste for Agricultural Application

**DOI:** 10.1021/acsomega.5c13053

**Published:** 2026-04-16

**Authors:** Thaís F. Rodrigues, Jenifer Panizzon, Marcia R. de Moura Aouada, Daniela M. de Quevedo, Vanusca D. Jahno

**Affiliations:** † Laboratory of Polymer Technology, Center for Research and Development in Clean Technologies, 125098 Feevale University, 2755 ERS-239, Novo Hamburgo 93525-075, Brazil; ‡ Hybrid Composites and Nanocomposites Group (GCNH), School of Engineering, 28108 São Paulo State University (UNESP), 56 Av. Brasil, Ilha Solteira 15385-000, Brazil; § Graduate Program in Environmental Quality, Feevale University, 2755 ERS-239, Novo Hamburgo, Rio Grande do Sul 93525-075, Brazil

## Abstract

Bananas and oranges are widely produced and consumed around the world, but peels and bagasse’s are considered inedible and are typically discarded. The development of bioplastics is one way to reuse this waste. This study aimed to produce biodegradable films from thermoplastic starch, banana peel and orange bagasse waste for use in soil mulching and temporary plant packaging. Soluble starch, glycerol and deionized water were used to prepare the films by casting, incorporating different blends of the fruit wastes previously dried and ground. Several analyses were performed on the fresh and powdered wastes and on the developed films. The results suggest the beginning of a degradation process in all films, without negatively influencing seed germination, which ranged from 57 to 100%. Application simulations were satisfactory. Our study demonstrates the benefits of recycling organic waste in the development of biodegradable films, promoting circularity and avoiding resource loss.

## 1 Introduction

The circular economy (CE) is a model that interrupts the logic based on extraction, transformation, consumption and disposal, with principles that promote waste and pollution elimination, avoiding wasting resources by keeping products and materials in use. It also recognizes that the goal is not to reduce impacts but to promote environmental quality, and considers the regeneration of natural systems, returning nutrients to the soil and ecosystems that need them.^1^ Fruits generate substantial amounts of waste that are separated into distinct parts, such as peels, leaves, stems, seeds, and others.^2^ Bananas and oranges are the most consumed and produced fruits in Brazil.^3^ In 2024, the production of bananas and oranges reached 7.05 and 15.7 million tons, respectively, destined for the domestic market and juice production.^4,5^ The banana peel represents approximately 35.2% of the fruit’s mass and is typically discarded.^6^ Oranges also generate significant waste (45–60% of the fruit) during consumption or juice processing.^7,8^ Given the significant volume of waste generated, the implementation of technological solutions is imperative to address this concern. This is particularly noteworthy when considering the potential of banana peel and orange bagasse, which are abundant in nutrients and organic compounds.

Carbon-rich organic compounds found in banana peel include cellulose (7.6–9.6%), hemicellulose (6.4–9.4%), pectin (10–21%), and lignin (6–12%). It is also rich in various minerals such as potassium, calcium, sodium, phosphorus, and magnesium, as well as trace elements (e.g., iron, copper, and manganese). However, these concentrations may differ depending on the variety, degree of maturation, and growing conditions such as soil type and rainfall.^9,10^ Orange waste contains several biopolymers and value-added compounds, mainly in the peel, consisting of soluble sugars (16.9%), starches (3.75%), fibers, including cellulose (9.21%), hemicellulose (10.5%), lignin (0.84%), and pectin (42.5%), as well as organic acids (e.g., citric acid, malic acid, malonic acid, oxalic acid, and ascorbic acid).^11^

A plastic material is defined as Bioplastic if it is biobased, biodegradable or has both properties.^12^ Biodegradable bioplastics must undergo significant microbial biodegradation for 180 days according to biodegradation standards (ASTM D5988 or EN 13432) and under favorable conditions such as temperature, pH, humidity, and others. As these plastics degrade, water, methane and/or CO_2_ and biomass are formed.^13^ Most bioplastics are considered first generation and are derived from carbohydrate-rich plants such as corn, sugar cane, castor beans, potatoes, or wheat. Second-generation bioplastics are made from nonfood crops, such as wood cellulose, and biomass processing waste, such as food or sawdust.^14^

In agriculture and horticulture, biodegradable polymers offer specific advantages, one of the most important examples being their use in mulching films, as there is no need to collect them after use. Other applications, such as plant pots for propagation and cultivation, are also considered promising. The packaging can thus be planted directly in the soil, as it quickly decomposes and allows the plant to grow.^15^ However, for biobased and biodegradable mulching films to comply with the regenerative principle proposed by the CE, it is necessary to use renewable raw materials and recover them through biological recycling, composting or biodegradation in the soil.^16^ Starch is a promising raw material due to its abundance and ability to be converted into thermoplastic starch (TPS).^17,18^ There are several methods of starch processing and the most commonly used is by casting. Nevertheless, its lack of scalability is a current research gap. One potential approach to industrial processing is continuous casting, which could facilitate the transition from laboratory-scale to industrial production in future research.^19^

In this context, the possibility of reusing fruit wastes, rich and abundant sources of polymers,^2^ occurs through the development of biodegradable films.^20,21^ The present study aims to develop biodegradable films based on thermoplastic starch using banana peel and orange bagasse waste for potential application as mulching films and temporary packaging for plants. It brings an innovative aspect presenting a comparative analysis of fruit waste mixtures in film formulation along with an integrated assessment of biodegradation, seed germination, and applied tests. This approach offers a solution that promotes the circularity of organic waste, allowing its components to return to the soil, providing a renewable alternative for agriculture.

## 2 Materials and Methods

Fresh Pome bananas (*Musa acuminata* × *Musa balbisiana*, AAB group) and Valencia oranges (*Citrus sinensis*) were obtained from local farmers (Taquara, Rio Grande do Sul, Brazil). Banana peels were visually classified as C7 according to the Von Loesecke ripeness scale, presenting a yellow color with brown spots.^22^ Sodium hypochlorite solution (NaClO 2.0 to 2.5% w/w) was used to sanitize the peels and bagasses. The polymeric matrix of the films was soluble starch P.A. ACS (Êxodo Científica, Brazil), and glycerol P.A. ACS (Labsynth, Brazil) as a plasticizer. Deionized water was used for all experiments.

### 2.1 Preparation of Banana Peel Powder (BPP) and Orange Bagasse Powder (OBP)

Banana peels (BP) and orange bagasses (OB) were sanitized separately in a solution of sodium hypochlorite (0.030–0.0375%) for 10 min. Blends of the waste were prepared as shown in Table 1. These blends were then ground using a food processor (800 W, Philco, Brazil) and subsequently dried in an oven (SL-101, Solab, Brazil) at 60 °C. To obtain the powders, the residues were ground a second time. Particles smaller than 0.5 mm were selected using a 32 mesh sieve (Bertel, Brazil). Particle size distribution is available in the Supporting Information (Figure S1).

**Table 1:** Blends of Powdered Waste: Banana Peel Powder (BPP); Orange Bagasse Powder (OBP)^a^

	blends (%)	
	BP	OB
**BPP**	100	0
**OBP**	0	100
**P**70/30	70	30
**P**30/70	30	70
**P**50/50	50	50

a BP and OB columns indicate the amount of fresh residue used for each blend.

The fresh waste generated after peeling the bananas and extracting the juice from the oranges was 35.67% for BP and 46.54% for OB, relative to the total weight of the fruit. The percentage of dry waste to fresh, by weight, was 8.19% for banana peel powder (BPP) and 15.56% for orange bagasse powder (OBP).

### 2.2 Preparation of Thermoplastic Starch and Biodegradable Films

Films were developed from 18 g starch and 5.4 g glycerol (30% m/m starch) in 600 mL of deionized water.^21^ A thermoplastic starch film was used as a control. The solution was maintained at 70 °C on a hot plate (Q261-22, Quimis, Brazil) with constant mechanical stirring (RW-20 digital, Ika, Germany) at 70 rpm for 30 min (Figure 1). Finally, the solution was equally divided into three 180 mL parts. Nonstick Teflon sheets were placed in 30 × 30 cm glass molds. The films were formed by the casting method in a bacteriological oven (SL-101, Solab, Brazil) at 60 °C for 8 h. Similarly, biodegradable films were produced by replacing 30% of the starch content with banana peel powder, orange bagasse powder and other blends.

**Figure 1:**
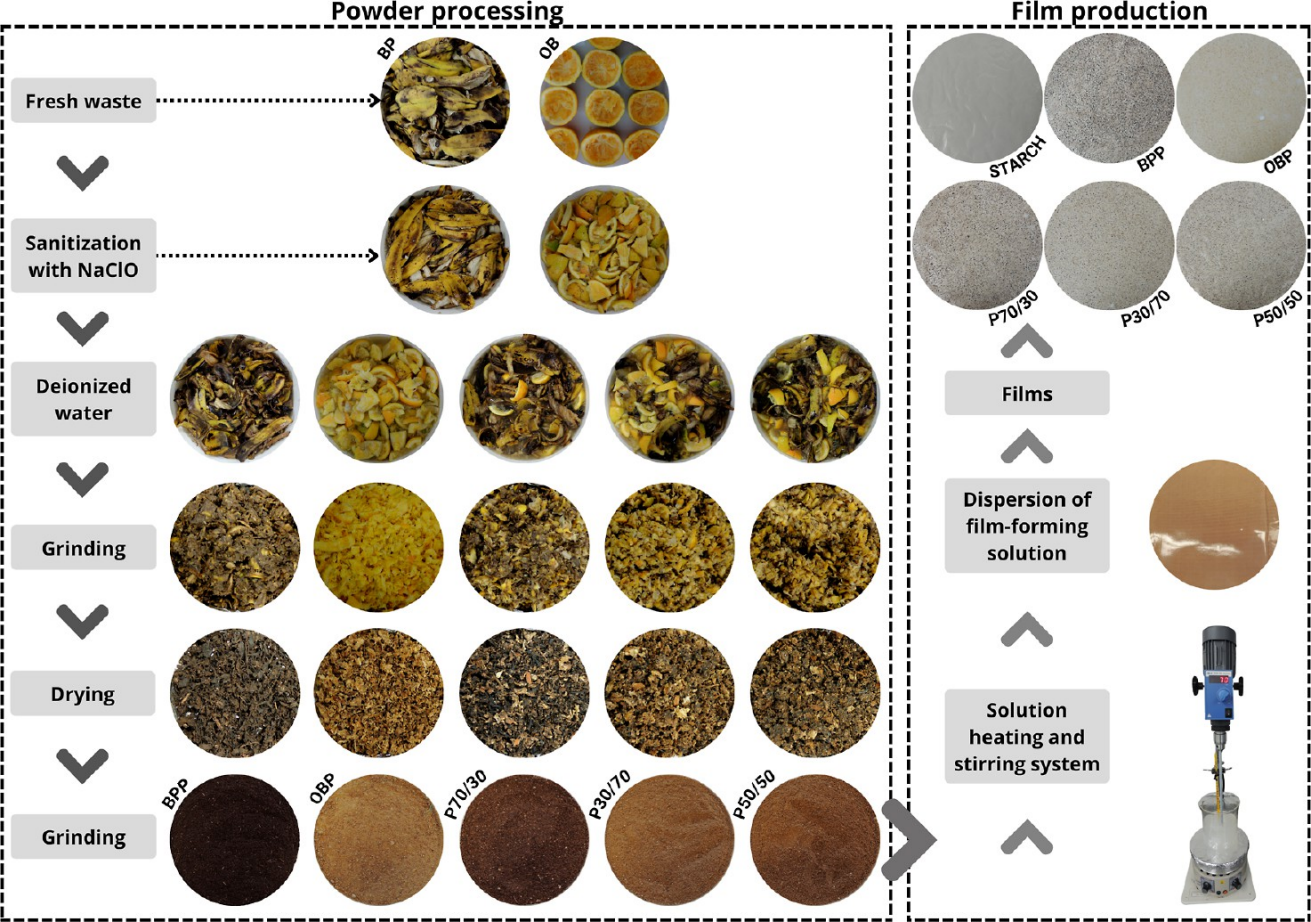
Schematic representation of powders processing and film preparation: banana peel (BP), orange bagasse (OB), banana peel powder (BPP), orange bagasse powder (OBP), 70% BP and 30% OB (P70/30), 30% BP and 70% OB (P30/70), and 50% BP and 50% OB (P50/50). Round colored image represents the solution prepared for the thermoplastic starch film used as a control and dispersed on nonstick Teflon sheets.

### 2.3 Fresh Waste, Powders and Film Characterization

#### 2.3.1 Visual Analysis

Photographs (Nikon Coolpix P510, China) were taken to evaluate the films visually.

#### 2.3.2 Thickness

Film thickness was measured using a digital micrometer (MDC-25SX, Mitutoyo, Japan) with 0.001 mm accuracy. The arithmetic mean of ten random measurements on different segments was reported for each film in triplicate.

#### 2.3.3 Scanning Electron Microscopy (SEM)

The surface and cross-sectional areas of the film samples were determined using a scanning electron microscope (JSM-6510LV, JEOL, Japan) at 10 kV. Micrographs were captured at 1000× magnification. For the cross-sectional area, the films were cryofractured in liquid nitrogen. Fruit waste powder (FWP) particles were observed at 500× magnification at 5 kV and 10 kV. Double-sided conductive carbon tape was used to attach the samples to cylindrical copper stubs. All samples were coated with a thin layer of gold for conductivity and kept in a desiccator until testing.

#### 2.3.4 Fourier Transform Infrared (FTIR) Spectroscopy

FTIR analysis of the FWP and the films was performed on a spectrophotometer (Frontier MIR, PerkinElmer, United Kingdom) equipped with an attenuated total reflection (ATR) accessory. For each ATR-FTIR spectrum (transmittance mode), 16 scans were accumulated with 4 cm^–1^ resolution in the 4000–650 cm^–1^ range.

#### 2.3.5 Mechanical Properties

Tensile strength and elongation at break were determined on a dynamometer (NS 354 software, Maqtest, Brazil), adapted from ASTM D882-09, with a load cell of 50 N, a speed of 50 mm min^–1^, and a distance between grips of 50 mm, using five specimens for each film. The samples were kept under controlled temperature and humidity conditions, at 22 °C and 50%, respectively.

#### 2.3.6 Thermogravimetric Analysis (TGA)

Thermal degradation profiles were obtained on a thermogravimetric analyzer (TGA 51H, Shimadzu, Japan). Using a synthetic air atmosphere at a flow rate of 60 mL min^–1^, samples of approximately 10 mg and 4 mg of the FWP and the films, respectively, were heated from 20 to 600 °C at a heating rate of 10 °C min^–1^.

#### 2.3.7 Moisture Content

A thermobalance (MB23, Ohaus, China) was used to determine the moisture content of FWP and the films, set to automatic operation until constant mass was reached, at a temperature of 105 °C. The analysis was performed in triplicate with 3 g samples.

#### 2.3.8 Preliminary Tests of Biodegradability and Seed Germination

For the biodegradation test, preliminary qualitative analyses were conducted.^23^ Samples of 25 mm^2^ films, in triplicate, were buried to a depth of 20 mm in commercial plant substrate (Carolina Soil, Brazil). Film samples in same size were also placed on the substrate. The test was conducted under controlled conditions (22 °C and 50% average relative humidity), and deionized water was sprayed 3 times during the week to maintain humidity. Commercial low-density polyethylene (LDPE) and cellulose films were tested as controls. Samples were collected at 7, 15, and 30 days for visual analysis with digital photographs and photomicrographs (Stemi 508, Zeiss, Germany). After removing the samples in the last period of the biodegradation test, seven black-eyed pea (*Phaseolus vulgaris*) seeds were planted per container and approximately 25 mL of water was added daily to maintain moisture for 14 days.^24^ The test was conducted at room temperature with sunlight coming through the windows. Subsequently, the plants were cleaned and their germination (%), growth (cm) and total biomass (g) were measured.

#### 2.3.9 Application Test

Based on visual analysis, starch and OBP films were selected to simulate the mulching and plant packaging applications proposed in this study. The films were placed on top of a substrate in rectangular pots containing three romaine lettuce (*Lactuca sativa*) seedlings each and remained for 40 days. The packaging was developed by sealing the sides and bottom of the film and planting a cravine flower (*Dianthus chinensis*) seedling inside.

#### 2.3.10 Simplified Cost Considerations

The calculation for producing 1 m^2^ of starch film considered the quantities of raw materials used, including soluble starch, glycerol, and deionized water, in addition to the consumed energy.

#### 2.3.11 Statistical Analysis

The results were expressed as means, their respective standard deviations, and medians. SPSS software (version 26) was used. Normality testing was performed using the Kolmogorov–Smirnov test. Since the data did not follow a normal distribution, nonparametric tests were applied for group comparisons. The Kruskal–Wallis test and pairwise comparisons were performed with a significance level of 5%.

## 3 Results and Discussion

### 3.1 Characterization of Fruit Waste Powder

The moisture content was significantly different between fresh and powered waste. The values obtained for BP and OB were higher than 80%, while BPP, OBP and blends ranged between 1.6 and 2.5% (see Table S1 in the Supporting Information for complete data). Fruit and vegetable fresh waste presents a high percentage of moisture, between 80 and 90%.^25^

The morphological analysis of the FWP obtained from the micrographs is shown in Figure 2. The BPP surface showed granules of uneven size and irregular shape, tending to elongate.^26^ Moreover, the surfaces were completely rough. This characteristic results from cell walls coating surfaces.^27^ The morphology observed was also similar to the banana peel and pulp blend,^28^ suggesting that the agglomerated surface may be related to the presence of B-type starch. In OBP, the particles are heterogeneous in size and show a rough surface,^29^ in addition to the agglomerated structures also observed.^30^

**Figure 2:**
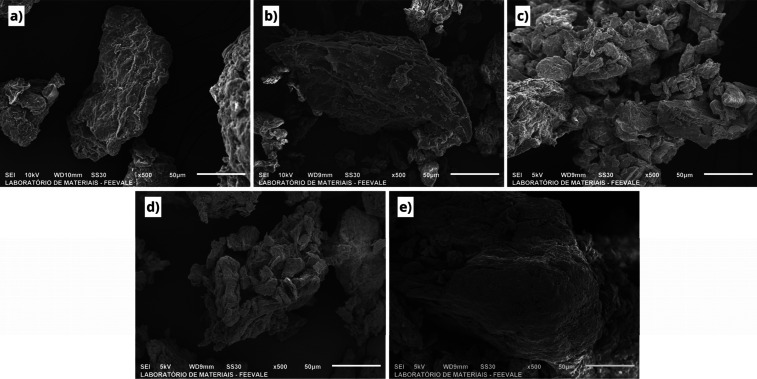
Scanning electron microscopy (SEM) images of powder surface morphology at 500× magnification: (a) BPP, (b) OBP, (c) P70/30, (d) P30/70 and (e) P50/50.

As illustrated in Figure 3, the chemical and thermal profile of the powdered wastes was analyzed through (a) FTIR spectra, (b) TGA curves, and (c) DTG curves. The chemical profile obtained from the FTIR spectra allows the identification of the main functional groups present in the powdered residues (Figure 3a). Broad absorption bands between 3304 and 3278 cm^–1^ correspond to the symmetrical and asymmetrical stretching of the OH groups, which are strongly present in phenolic compounds in BPP and generally present in carbohydrates and lignin in OBP. The bands between 3000 and 2800 cm^–1^ can be attributed to the stretching of the hydrogen in the CH bond, such as the CH_2_ and CH_3_ groups. The band at 1736 cm^–1^ is associated with carbonyl groups (esters). The band around 1600 cm^–1^ indicates the presence of aromatic compounds in the C=C stretch, related to orange peel. The protein fractions present in banana peel, such as albumin, globulin, prolamin and glutelin, show peaks between 1690 and 1229 cm^–1^, indicating the presence of amide I, amide II or amide III, but at 1564 cm^–1^ the vibration resembles the CN stretching and NH bending of amide II. The band at 1011 cm^–1^ is associated with the alcohol or ester group and forms the basic structure of lignocellulosic materials such as orange peel. At 1023 cm^–1^, the bending vibrations of the COH bond are attributed to the starch molecule.^31–35^

**Figure 3:**
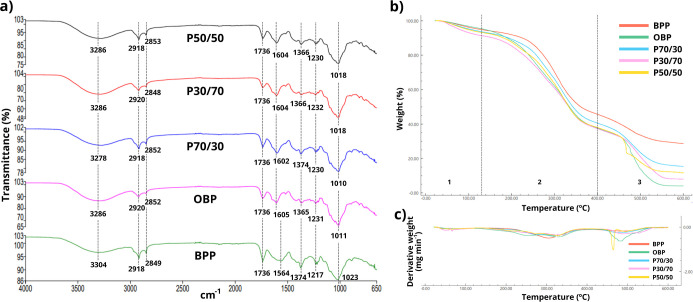
Analysis of the chemical and thermal profile of the powdered wastes: (a) FTIR spectra, (b) TGA curves and (c) DTG curves.

TGA (Figure 3b) and derivative thermogravimetric (DTG) curves (Figure 3c) indicate that there were three stages of mass loss: (1) between 63.8 and 72.9 °C; (2) between 278.1 and 294.4 °C; (3) between 467.0 and 498.4 °C. In the first stage, a maximum mass loss of 7.8% was attributed to moisture and volatile compounds.^36–38^ In the second stage, the thermal decomposition of the starch macromolecules occurred.^39^ This was the moment when the greatest mass loss occurred in all powdered waste (between 46 and 57%), results from the release of volatile degradation products generated during chain scission (H_2_O, CO_2_, CO, and low-molecular-weight oxygenated compounds). FWPs are rich in proteins, lipids, carbohydrates and fiber. Since they occur simultaneously, it is difficult to determine an exact mass loss temperature for each fraction. Therefore, the decomposition of these components can also be attributed to mass loss at these temperatures.^40^

Additionally, cellulose decomposition may occur between 220 and 400 °C,^35^ while protein or oxidation of partially decomposed proteins occurs between 200 and 500 °C.^41^ The carbonization and the formation of fixed mineral residues from organic matter can be attributed to the last thermal event, with a mass loss of between 14 and 30%.^36,40^ Hence, at the end of the analysis, the remaining mass consists of inorganic material such as ash.^41^ In this last stage, P50/50 showed a different mass loss curve profile than the others at 467 °C, referring to the degradation of lignin in the last stage of biomass, such as orange peel.^42^ Data regarding mass loss and peak degradation temperatures for each FWP can be found in Table S2 of the Supporting Information.

### 3.2 Characterization of Starch-Based Films with Fruit Powder Waste

#### 3.2.1 Visual Analysis

Images in Figure 4 show that the tonalities of the films varied. The banana peel films were brown in color, darker and less transparent than the starch film.^20^ In contrast, OBP films showed a yellow tone, which is associated with the presence of carotenoids from orange waste.^43^

**Figure 4:**
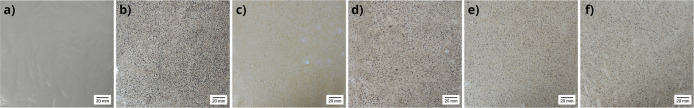
Films surface photographs: (a) starch film, (b) BPP film, (c) OBP film, (d) P70/30 film, (e) P30/70 film and (f) P50/50 film.

#### 3.2.2 Morphological Characteristics

The FWP did not dissolve in the starch solution. Consequently, the particles were visible on the surface and inside the film, as observed for the OBP film in Figure 5. Microcracks were visible, but less in the starch film. This may be attributed to the properties of the materials and the casting process used to prepare the films, or the coating layer added when preparing the samples for analysis.^23,44^ Nevertheless, the homogeneous structure indicates that there were no distinct phases in the film matrix.

**Figure 5:**
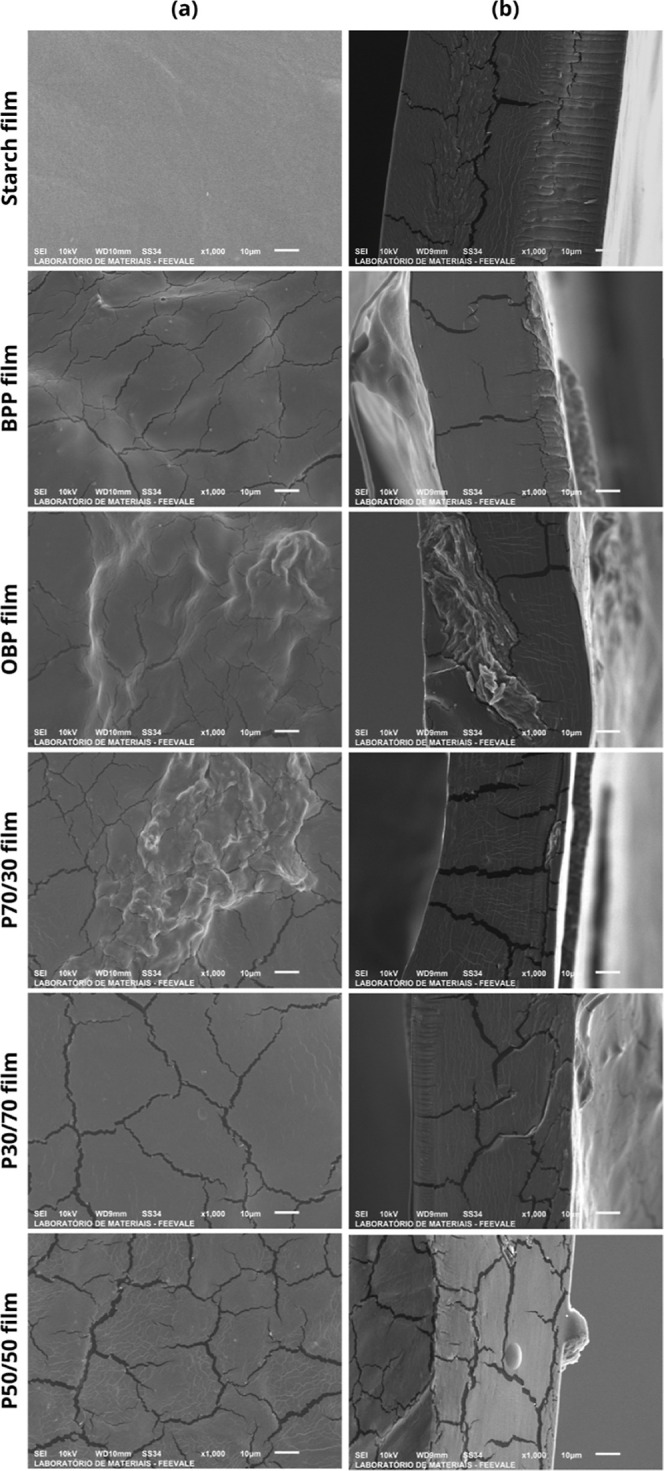
Scanning electron microscopy (SEM) images of (a) surface morphology and (b) cross-section of the films at 1000× magnification.

#### 3.2.3 Characterization by Fourier Transform Infrared (FTIR) Spectroscopy

The chemical structure profile of the biodegradable films obtained from the spectra was predominantly related to starch (Figure 6a).

**Figure 6:**
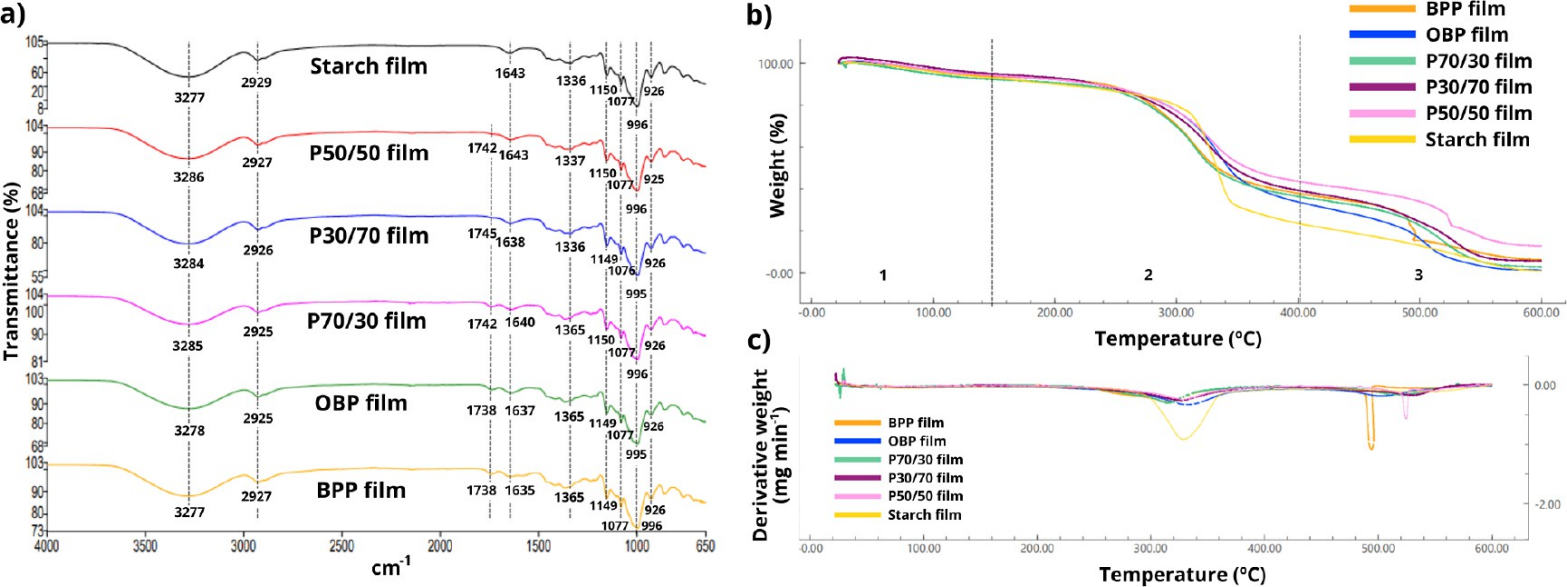
Analysis of the chemical and thermal profile of the films: (a) FTIR spectra, (b) TGA curves and (c) DTG curves.

Between 3500 and 3000 cm^–1^, the OH stretching band was assigned. This band also showed a higher vibration intensity due to the presence of hydroxyl groups from the use of glycerol as a plasticizer. The band at 2929 cm^–1^ corresponds to the CH_2_ stretching bonds. The bending vibration related to the H–O–H bond of water occurred at 1643 cm^–1^. The bands at 1336 cm^–1^, 1150 cm^–1^ and 1077 cm^–1^ correspond to the bending vibrations of the C–O–H bond, C–O–H stretching and C–O–H stretching, respectively, and the C–O–C stretching at 996 cm^–1^, which are characteristic of starch. The absorption at 926 cm^–1^ indicated the presence of an α-1, 6-D glycosidic linkage.^23,45–47^ The bands at 1745 cm^–1^, 1742 cm^–1^ and 1738 cm^–1^ indicate a difference in the spectra of the fruit waste films compared to the starch film. This stronger absorption region may be related to the chemical structure of the FWP particles, as shown in Figure 3a.

#### 3.2.4 Thermogravimetric Analysis (TGA)

The characterization of thermal properties is essential to assess stability and optimize processing conditions, including scalability for industrial use.^19^ Three stages of mass loss in the biodegradable films are shown: (1) between 71.8 and 82.6 °C; (2) between 304.6 and 325.3 °C; (3) between 492.5 and 531.0 °C (Figure 6b,c). The first thermal event corresponded to the loss mass of free and bound water and low-molecular-weight volatiles (between 3.7 and 7.4%). However, moisture loss is not observed exclusively in this thermal event, especially considering the presence of bound water associated with starch, glycerol, and lignocellulosic components. This was followed by a stabilization stage in all samples. In the second thermal event, the highest percentage of mass loss in the films was obtained, especially in the starch film, with 64%, related to the decomposition of the glycerol-rich phase. The last event was attributed to starch thermal decomposition, corresponding mainly to the polyhydroxyl groups elimination.^48^ Consistent with the chemical structure analysis, the similarity of the thermogravimetric results compared to the starch film, indicates that the properties of starch were predominant in the structure of the film, as it is the polymer with the highest mass content.^23^ Refer to Table S3 of the Supporting Information for data on mass loss and peak degradation temperatures for each film.

#### 3.2.5 Thickness and Moisture Content of Films

As shown in Table 2, the starch film had a significantly different thickness when compared to the other films containing FWP. This is a result of increasing film solids.^20^ Another influencing factor is viscosity, as more liquid solutions form thinner films.^49^ The moisture content (Table 2) showed no significant difference between the films with FWP, however, the starch film showed the highest percentage.^50^ This behavior is associated with microstructure and crystallinity, considering that water may be present in both the amorphous phase (entrapped within pores) and the crystalline phase of starch films.^51^

**Table 2:** Thickness, Moisture Content and Mechanical Properties of Biodegradable Films^a^

	thickness (mm)		moisture content (%)	
sample	mean and SD	median	mean and SD	median
starch film	0.11 ± 0.02	0.11^c^	15.00 ± 0.78	15.4^a^
BPP film	0.29 ± 0.04	0.29^ab^	11.13 ± 0.47	11.3^b^
OBP film	0.28 ± 0.04	0.28^b^	13.43 ± 0.06	13.4^ab^
P70/30film	0.32 ± 0.04	0.32^a^	14.57 ± 0.95	14.9^ab^
P30/70film	0.31 ± 0.04	0.31^ab^	13.83 ± 1.46	14.0^ab^
P50/50film	0.32 ± 0.04	0.32^a^	12.97 ± 1.10	12.6^ab^

	tensile strength (MPa)		elongation at break (%)	
sample	mean and SD	median	mean and SD	median
starch film	4.48 ± 0.43	4.56^a^	26.70 ± 4.08	24.68^a^
BPP film	0.79 ± 0.13	0.79^b^	8.66 ± 3.63	8.12^b^
OBP film	1.62 ± 0.73	1.61^ab^	14.19 ± 6.59	15.20^ab^
P70/30 film	1.19 ± 0.43	1.03^ab^	8.05 ± 3.27	7.78^b^
P30/70 film	1.18 ± 0.14	1.22^ab^	10.28 ± 3.55	10.48^b^
P50/50 film	0.94 ± 0.16	0.88^b^	13.25 ± 2.29	12.50^ab^

a Note: Banana peel powder (BPP); Orange bagasse powder (OBP) and Standard deviation (SD). ^a–c^ Different letters in the same column indicate significant difference using the Kruskal–Wallis test (*p* < 0.05).

#### 3.2.6 Mechanical Properties

Incorporating BPP significantly reduced the tensile strength and elongation of the film compared to the starch-based film.^20^ In films containing BPP, tensile strength is significantly affected by starch concentration, whereas elongation is significantly affected by heating time.^49^ It is also possible that no interfacial adhesion occurred between the starch matrix and the powder because an increase in tensile strength is expected with the addition of fillers.^52^ There was no significant difference between the evaluated parameters in the films with powder residues (Table 2). Additionally, the mechanical integrity of the films may be compromised due to starch’s hydrophilicity, as amylose and amylopectin contain numerous hydroxyl groups that can form hydrogen bonds with water molecules.^53^ Starch-based films are typically semicrystalline, because starch chains can reassociate during drying and storage (retrogradation), generating ordered domains associated with double-helical organization embedded within an amorphous fraction that is strongly influenced by water and glycerol plasticization.^54,55^ The relative contribution of ordered versus amorphous fractions depends on the amylose/amylopectin architecture and on plasticizer/water content and storage conditions: amylose-rich systems tend to develop ordered domains more readily, while amylopectin retrogrades more slowly, often remaining less ordered/more amorphous under typical casting times and early storage.^55,56^ In general, a higher fraction/continuity of ordered domains tends to increase stiffness and tensile strength at the expense of ductility, whereas a higher amorphous/plasticized fraction tends to favor elongation but can reduce strength.^54,57^ Importantly, in particle-filled systems (films containing fruit-waste powders), the mechanical response also depends strongly on particle dispersion and interfacial adhesion; coarse particles and interfacial gaps act as microstructural discontinuities that can initiate failure and reduce both tensile strength and elongation even if changes in structural order are modest.^54,58^ Because X-ray diffraction analysis (XRD) were not performed in this study, we do not quantify crystallinity; instead, we discuss these mechanisms qualitatively and highlight XRD as priority future work to track retrogradation-related structural evolution.^54,55^ As a limitation of the study, a reduction in the particle size of FWP should be considered for future studies. However, in the context of short-term, biodegradable agricultural applications, rapid degradation may be prioritized over mechanical strength.

#### 3.2.7 Preliminary Tests of Biodegradability and Seed Germination

No fragmentation was observed in the films placed on the plant substrate during the evaluation period, but samples did curl due to moisture, particularly the starch film. Color changes were observed in all buried films. The samples became lighter in color, opaque, fragmented, rough in texture, and covered with substrate particles (Figure 7). The qualitative assessment of biodegradability showed that the observation of cracks, in addition to considerable changes in color and tone, suggests the occurrence of a degradation process, which is also directly related to the hydrophilic properties of the material.^21^ Strong changes in tone and integrity indicate the beginning of degradation,^59^ and that a progressive change in the surface of the samples was observed, including cracking, holes, color changes and the appearance of microorganisms.^60^ For quantitative analysis, samples were weighed prior to testing, but biodegradation measurements by mass loss of bioplastics are extremely difficult to perform^61^ due to substrate adhesion to the film surface.

**Figure 7:**
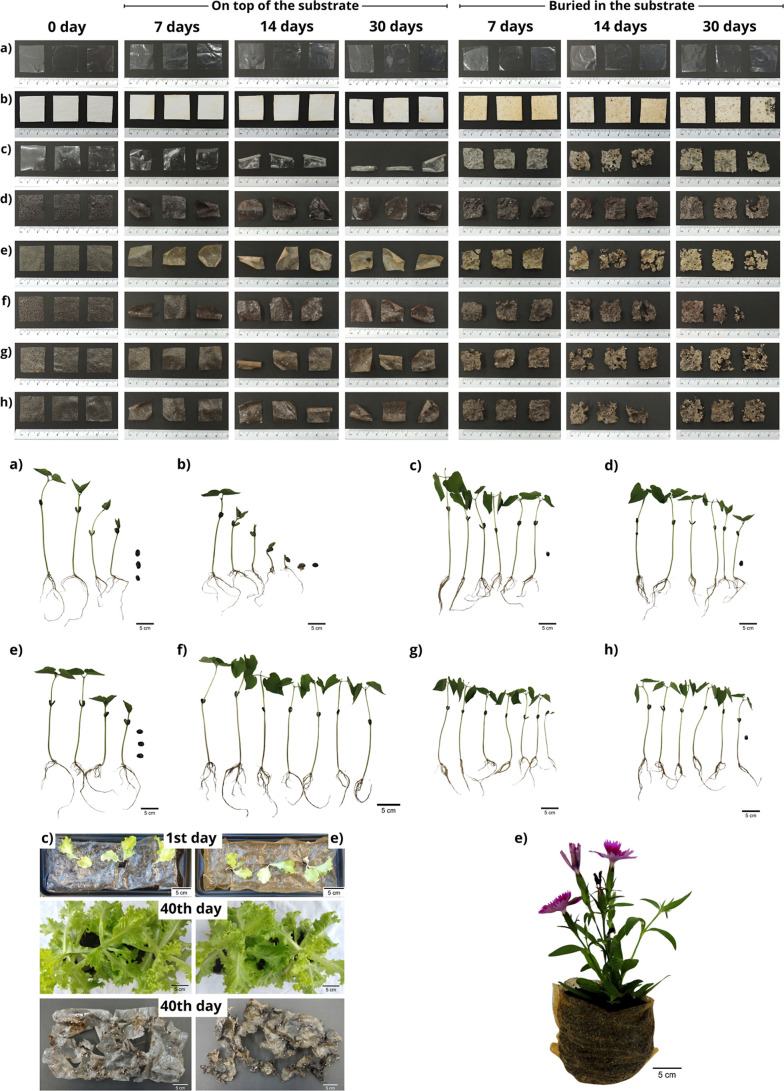
Biodegradation of developed films, germinated seeds and applications as mulching films and plant packaging: (a) LDPE, (b) cellulose, (c) starch film, (d) BPP film, (e) OBP film, (f) P70/30 film, (g) P30/70 film and (h) P50/50 film.

Seed germination was used to verify the condition of the substrate in which the samples were buried for 30 days. The potential toxicity of the soil was investigated because during the biodegradation process, polymers can release substances that affect plant germination and growth.^24^ Figure 7 shows the development of plants. More data related to this analysis can be found in Tables S4 and S5 of the Supporting Information.

The biomass and shoot growth of plants germinated in the starch film substrate showed higher values and were significantly different from those of LDPE and cellulose. No significant difference was observed for root growth. Depending on the quality and quantity of nutrients and/or contaminants in the soil used, the difference in biomass may be influenced by several factors, such as seed quality, germination time, solar incidence (morning/afternoon), film degradation, and plant growth.^24,62^ Germination rates varied from 57 to 100%, without significant correlation. These findings under the tested conditions suggest that the film composition does not cause adverse effects, enabling this plant species to grow and develop.^23^

#### 3.2.8 Performance Analysis of Mulch Film and Packaging for Plants

OBP film and starch film were selected to simulate mulch film application in romaine lettuce growing, as shown in Figure 7 (see Figure S2 in the Supporting Information for seedling development). After 40 days, the films showed color changes and dark spots, a wrinkled and twisted appearance, in addition to cracking and fragmentation in both materials. These aspects were more intense than those observed in the films used in the biodegradation test (available in Figures S3 and S4 of the Supporting Information), due to the deposition of organic matter from the dried leaves on the material, along with greater water requirement for seedling growth. In the second application, a prototype was developed as a temporary packaging for plants, and the flower was transplanted from its original packaging to the developed prototype (Figure 7). The proposed simulations reinforce the validation of the biodegradation and germination tests. As expected, the films underwent an initial degradation process due to their interaction with water molecules and their formulation containing starch and FWP. This process did not negatively impact the development of the tested plants.

Although the simulation suggests a potential agricultural application, economic feasibility must be assessed to determine its applicability in real production and marketing contexts. Regarding laboratory-scale costs, the production of 1 m^2^ of starch film was estimated to cost approximately 3.84 dollars. Studies on this topic are scarce, and the available literature estimates costs to be much higher than those of petroleum-derived plastic films. Still, current data is insufficient for a comprehensive comparison of biobased and fossil-based plastics from an innovative bioeconomy perspective.^63,64^

## 4 Conclusions

Our study addresses the circularity of organic waste by performing a comparative analysis of FWP blends in films and an integrated evaluation of biodegradation, seed germination, and applied tests. Considering the Brazilian agro-industrial context, bananas and oranges stand out as the most produced fruits while generating millions of tons of organic waste after consumption. Managing this waste presents several structural and operational challenges, often resulting in improper disposal in landfills and dumps, which occupies large areas and poses a risk of environmental contamination.

In this scenario, it is essential to propose technologies that enable reuse. Organic waste from domestic and industrial sources, particularly banana peel and orange bagasse, can be relevant for developing biodegradable thermoplastic starch films with potential applications in agriculture. Processing of inedible parts allowed for better conservation and delayed natural degradation due to the significant reduction in moisture content between fresh and dry waste.

Using different FWP formulations to produce films demonstrated the feasibility of recycling fruit waste, resulting in materials with characteristic brown and yellow tones, greater thickness, lower moisture content, and high thermal stability. Preliminary testing suggests the beginning of a degradation process in 30 days and, in all films, no evidence of toxicity in seed germination was observed. Simulated applications in the agricultural sector include use as mulching films and as temporary packaging for plants, offering a renewable and regenerative alternative to an industry sector that is still heavily dependent on fossil-based plastics.

## Supplementary Material



## Notes

Particle size distribution, moisture content in fresh and powdered waste, thermogravimetric analysis and germination data. In addition to supplementary images of the application as mulch films and biodegradation tests (PDF)
