# Supplementary material for: Biodegradable Starch-Based Films Incorporating Banana and Orange Waste for Agricultural Application
Source: ACS Omega. 2026 Apr 16;11(16):24202–12. doi: 10.1021/acsomega.5c13053 (PMC13130103; doi:10.1021/acsomega.5c13053)

# Biodegradable starch-based films incorporating banana and orange waste for agricultural application

Thaís F. Rodrigues [a], Jenifer Panizzon [a], Marcia R. de Moura Aouada [b], Daniela M. de Quevedo [c], Vanusca D. Jahno [a,c*]

[a] *Laboratory of Polymer Technology, Center for Research and Development in Clean Technologies, Feevale University, 2755 ERS-239, Novo Hamburgo, 93525-075, Brazil.*

[b] *Hybrid Composites and Nanocomposites Group (GCNH), School of Engineering, São Paulo State University (UNESP), 56 Av. Brasil, Ilha Solteira, 15385-000, Brazil.*

[c] *Graduate Program in Environmental Quality, Feevale University, 2755 ERS-239, Novo Hamburgo, Rio Grande do Sul, 93525-075, Brazil.*

* E-mail: vanusca@feevale.br

**Figure S1 – Particle size distribution.**

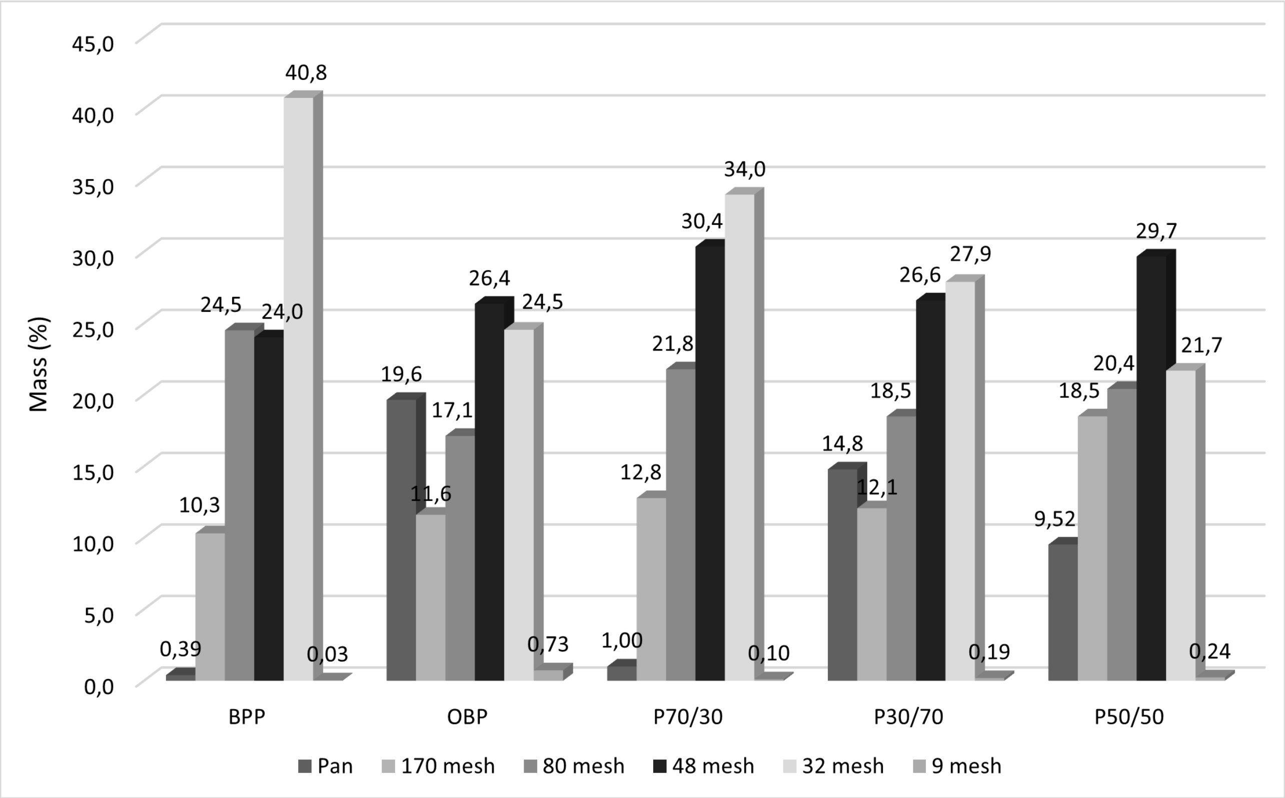

**Table S1 – Moisture content of fresh and powdered waste.**

| | Moisture content (%) | |
|---|---|---|
| | **Mean and SD** | **Median** |
| **BP** | 83.47 ± 0.67 | 83.80 [a] |
| **OB** | 81.13 ± 1.05 | 81.10 [ab] |
| **BPP** | 2.00 ± 0.30 | 2.00 [ab] |
| **OBP** | 1.97 ± 0.35 | 2.00 [ab] |
| **P70/30** | 2.50 ± 0.17 | 2.60 [ab] |
| **P30/70** | 1.77 ± 0.21 | 1.70 [ab] |
| **P50/50** | 1.60 ± 0.30 | 1.60 [b] |

**Table S2 – Thermogravimetric Analysis.**

| Sample | Weight loss (%) | T onset (ºC) | Tp (ºC) |
|---|---|---|---|
| BPP | 4.75 | 47.1 | 72.9 |
| | 45.95 | 252.7 | 294.4 |
| | 14.09 | 448.8 | 474.0 |
| OBP | 3.75 | 44.7 | 69.4 |
| | 57.37 | 219.9 | 278.1 |
| | 29.95 | 459.5 | 487.4 |
| P70/30 | 4.82 | 47.2 | 67.8 |
| | 50.88 | 244.5 | 293.1 |
| | 22.14 | 452.0 | 483.7 |
| P30/70 | 7.78 | 47.8 | 63.8 |
| | 52.38 | 234.3 | 281.7 |
| | 25.58 | 466.4 | 498.4 |
| P50/50 | 6.82 | 41.0 | 69.4 |
| | 52.11 | 244.6 | 288.1 |
| | 22.23 | 459.5 | 467.0 |

**Table S3 – Thermogravimetric Analysis.**

| Sample | Weight loss (%) | T onset (ºC) | Tp (ºC) |
|---|---|---|---|
| Starch film | 4.02 | 54.6 | 72.8 |
| | 64.37 | 294.2 | 325.3 |
| | 12.37 | 504.8 | 531.0 |
| BPP film | 3.72 | 63.3 | 77.2 |
| | 52.43 | 286.8 | 304.6 |
| | 25.90 | 490.1 | 492.5 |
| OBP film | 4.81 | 57.3 | 82.6 |
| | 60.27 | 285.5 | 321.5 |
| | 26.94 | 473.9 | 500.7 |
| P70/30 film | 7.39 | 41.7 | 71.8 |
| | 55.14 | 282.0 | 305.4 |
| | 26.99 | 492.3 | 513.5 |
| P30/70 film | 6.66 | 57.5 | 78.3 |
| | 54.50 | 286.8 | 313.6 |
| | 27.49 | 497.3 | 514.9 |
| P50/50 film | 5.95 | 59.1 | 77.9 |
| | 49.53 | 287.9 | 317.1 |
| | 24.62 | 515.2 | 522.4 |

**Table S4 – Bean seed germination test: plant biomass, shoot growth, and root growth, for all samples.**

| | Biomass (g) | | Shoot growth (cm) | | Root growth (cm) | |
|---|---|---|---|---|---|---|
| | Mean and SD | Median | Mean and SD | Median | Mean and SD | Median |
| **LDPE** | 1.23 ± 0.28 | 1.22 [b] | 17.0 ± 4.1 | 17.65 [bc] | 14.9 ± 2.7 | 15.45 [a] |
| **Cellulose** | 1.02 ± 0.25 | 0.97 [b] | 8.2 ± 6.2 | 7.20 [c] | 10.5 ± 5.0 | 11.40 [a] |
| **Starch film** | 2.05 ± 0.19 | 2.03 [a] | 24.7 ± 2.0 | 24.25 [a] | 14.9 ± 2.0 | 15.00 [a] |
| **BPP film** | 1.69 ± 0,24 | 1.67 [ab] | 21.8 ± 2.3 | 22.80 [abc] | 13.4 ± 1.8 | 13.45 [a] |
| **OBP film** | 1.35 ± 0.13 | 1.32 [ab] | 16.5 ± 3.4 | 16.75 [bc] | 15.2 ± 1.2 | 14.75 [a] |
| **P70/30 film** | 1.65 ± 0.27 | 1.66 [ab] | 23.6 ± 1.4 | 23.00 [ab] | 14.5 ± 2.9 | 14.50 [a] |
| **P30/70 film** | 1.69 ± 0.42 | 1.87 [ab] | 21.9 ± 2.7 | 22.20 [abc] | 13.7 ± 1.9 | 13.40 [a] |
| **P50/50 film** | 1.75 ± 0.27 | 1.70 [ab] | 22.4 ± 0.9 | 22.35 [abc] | 13.3 ± 2.8 | 13.10 [a] |

**Table S5 – Bean seed germination rate for all samples.**

| | Germination (%) |
|---|---|
| **LDPE** | 57 |
| **Cellulose** | 86 |
| **Starch film** | 86 |
| **BPP film** | 86 |
| **OBP film** | 57 |
| **P70/30 film** | 100 |
| **P30/70 film** | 100 |
| **P50/50 film** | 86 |

**Figure S2 – Application as mulching films: a) Starch film and b) OBP film.**

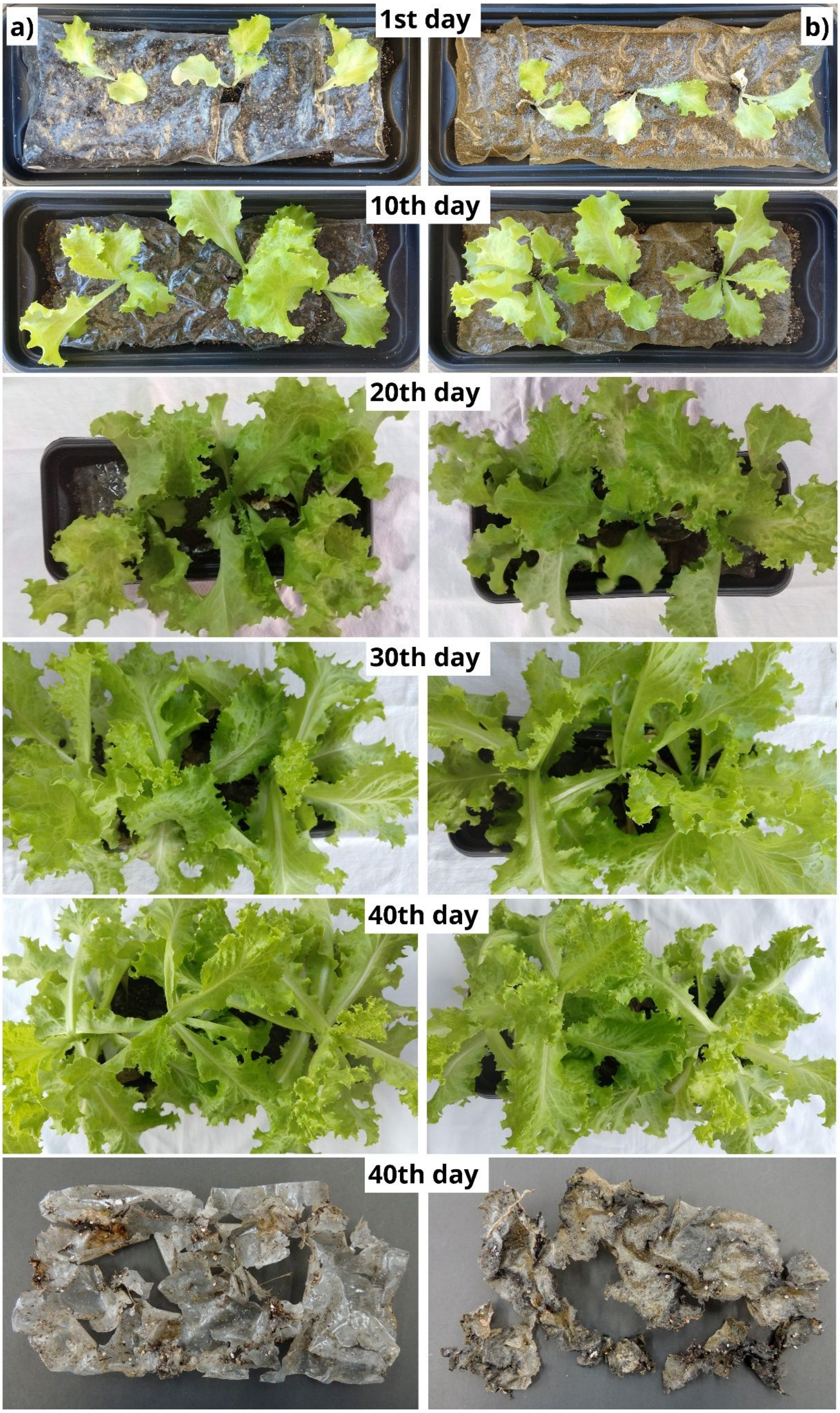

**Figure S3 – Application as mulching film after 40 days at 25.6x magnification: a) Starch film and b) OBP film.**

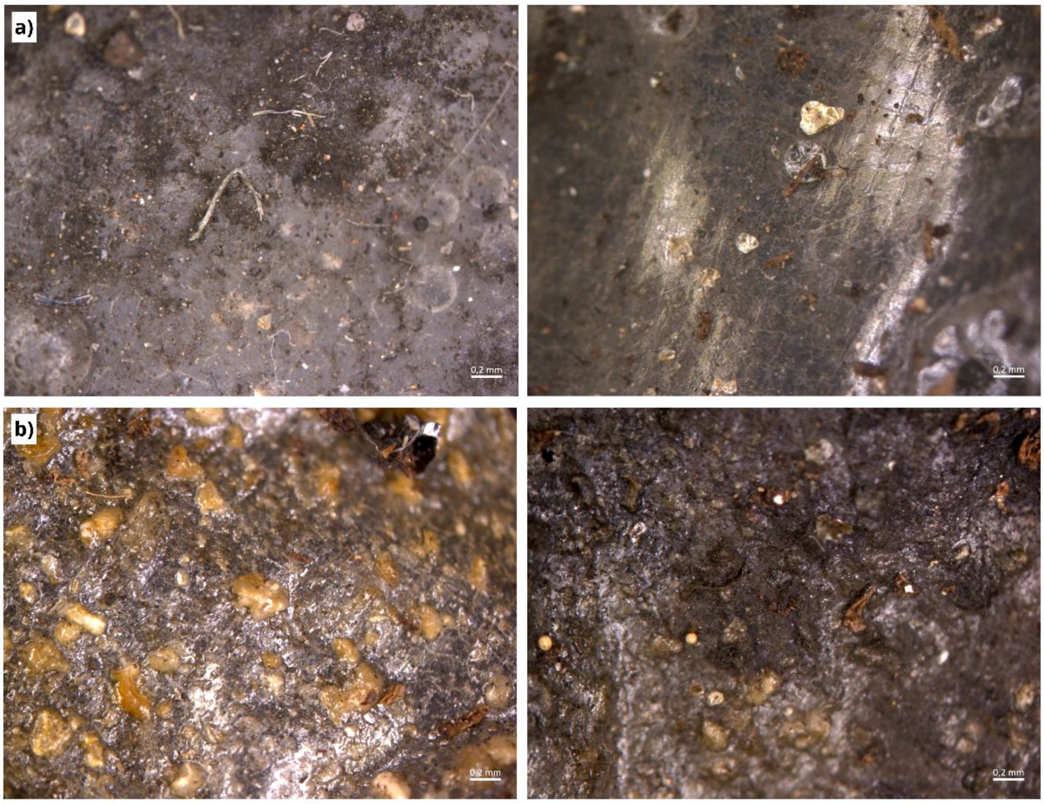

**Figure S4 – Biodegradation of films at 25.6x magnification on top of substrate (a, c) and buried on the substrate (b, d): Starch film (a, b) and OBP film (c, d).**

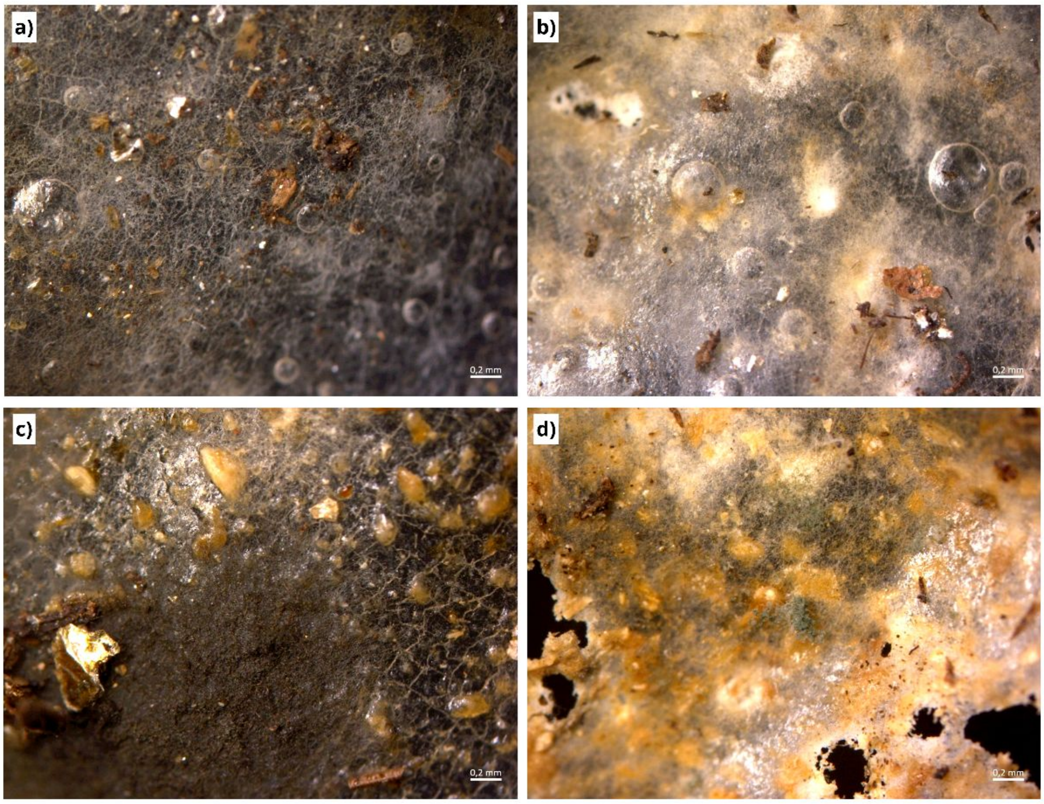

Supplement: Supplementary file 1 [file ao5c13053_si_001.pdf]
